# Identification of Rare Mutations of Two Presynaptic Cytomatrix Genes *BSN* and *PCLO* in Schizophrenia and Bipolar Disorder

**DOI:** 10.3390/jpm11111057

**Published:** 2021-10-21

**Authors:** Chia-Hsiang Chen, Yu-Shu Huang, Ding-Lieh Liao, Cheng-Yi Huang, Chia-Heng Lin, Ting-Hsuan Fang

**Affiliations:** 1 Department of Psychiatry, Chang Gung Memorial Hospital-Linkou, Taoyuan 333, Taiwan; yushuhuang1212@gmail.com; 2 Department and Institute of Biomedical Sciences, Chang Gung University, Taoyuan 333, Taiwan; genie.cgu@gmail.com; 3 Taoyuan Psychiatric Center, Department of General Psychiatry, Taoyuan 330, Taiwan; dlliao@typc.mohw.gov.tw (D.-L.L.); chlinakb@typc.mohw.gov.tw (C.-H.L.); 4 Bali Psychiatric Center, Department of Community Psychiatry, New Taipei City 249, Taiwan; tom@balipc.gov.tw

**Keywords:** schizophrenia, bipolar disorder, presynaptic cytomatrix, active zone, neurotransmission

## Abstract

Schizophrenia and bipolar disorder are severe mental disorders with a major component of genetic factors in their etiology. Rare mutations play a significant role in these two disorders, and they are highly heterogeneous and personalized. Identification of personalized mutations is essential for the establishment of molecular diagnosis, providing insight into pathogenesis and guiding the personalized treatment for each affected patient. We conducted whole-genome sequencing analysis of families with schizophrenia and bipolar disorder to search for their genetic underpinnings. This report identified a rare missense mutation Arg1087Gln of *BSN* (bassoon presynaptic cytomatrix protein) co-segregating with schizophrenia in a family with multiple affected members. Furthermore, we identified the rare missense mutation Ser1535Leu of *PCLO* (piccolo presynaptic cytomatrix protein) in two sisters with bipolar disorder and another rare missense mutation, His5142Arg in *PCLO*, in a patient with schizophrenia. These three missense mutations were very rare and were predicted to be pathogenic. The *BSN* and *PCLO* genes encode two structurally related proteins of the presynaptic cytomatrix at the active zone that regulates neurotransmission at the presynaptic neuronal terminal. Our findings suggest the involvement of the presynaptic matrix in the pathogenesis of schizophrenia and bipolar disorder, and *BSN* and *PCLO* are the risk genes for schizophrenia and bipolar disorder.

## 1. Introduction

Schizophrenia and bipolar disorder are two severe psychiatric disorders with a significant genetic component in their etiology [1,2]. These two disorders’ genetic bases are highly heterogeneous, including many common SNPs and rare mutations. A significant overlap of the genetic variants between schizophrenia and bipolar disorder suggests that these disorders share some common heritability [3,4,5]. The advancements of chromosomal microarray analysis and next-generation sequencing (NGS) technologies have facilitated the detection of rare pathogenic mutations in patients with psychiatric disorders. Accumulating evidence indicates that rare mutations associated with psychiatric disorders are highly heterogenous and personalized. Identifying personalized mutations can help establish the molecular diagnosis, elucidate pathogenesis, and guide personalized treatment for affected patients.

The release of neurotransmitters from the presynaptic neuronal terminal is a complicated process. The active zone (AZ) is a specialized area of the presynaptic terminal membrane where neurotransmission occurs. The AZ area is characterized by the presence of the electron-dense protein meshwork–presynaptic cytomatrix (PCM). The PCM is a very complex structure consisting of several multi-domain scaffolding proteins, including Rab3-interacting molecules (RIMs), RIM-binding proteins (RIM-BPs), Bassoon and Piccolo/Aczonin, CAST/ELKS/Bruchpilot proteins, Liprins-α, and UNC-13/Munc-13 proteins [6,7,8]. Each protein has its unique function in regulating neurotransmission [6,9,10,11,12]. Furthermore, several studies indicate that the PCM functions as a regulator of neurotransmission and involves synaptogenesis, synapse specificity, and synaptic plasticity [8,13].

The *BSN* gene is located at 3p21.31 [14,15,16], while the *PCLO* gene is located at chromosome 7q21.11 [17]. These two genes encode bassoon presynaptic cytomatrix protein and piccolo presynaptic cytomatrix protein, respectively. They are structurally related because they have high homology of protein sequences, including two zinc finger motifs and three potential coiled-coil regions [6]. Bassoon and piccolo proteins are multifunctional proteins and play a critical role in regulating the assembly of AZ and the trafficking, docking, release, and recycling of synaptic vesicles [12,18]. Moreover, these two proteins are involved in the maintenance of the integrity of AZ [12]. Furthermore, both proteins are associated with activity-dependent neuronal plasticity, including short- and long-term plasticities [19,20].

Mutations of *BSN* have been implicated in neurodevelopmental and neurodegenerative disorders. A recent study reported the discovery of several missense mutations of *BSN* in patients with the familial and sporadic type of progressive supranuclear palsy-like syndrome, a rare neurodegenerative disorder of tauopathy with characteristic neuropathological findings in the brain [21]. Patients with multiple system atrophy, a rare progressive neurodegenerative disease, have been found to have increased *BSN* transcript expression in their postmortem brains compared to controls [14]. Additionally, *BSN* is considered a candidate in the 3p21.31 microdeletion syndrome, a rare disease characterized by developmental delay and distinctive facial features [22].

Several studies have indicated that *PCLO* is a risk gene for affective disorder. The single-nucleotide polymorphism (SNP) rs2522833 of *PCLO* has been reported to be associated with major depressive disorder in several studies [23,24,25,26]. In contrast, another SNP, rs13438494, of *PCLO* is associated with bipolar disorder in a meta-analysis of the genome-wide association studies (GWASs) of bipolar disorder [27]. Additionally, the SNP rs13438494 of *PCLO* is associated with drug abuse [28]. Furthermore, a rare homozygous nonsense mutation has been found in a family with pontocerebellar hypoplasia type III [29], and deletion of the *PCLO* gene is thought to be involved in patients with intellectual disability [30]. Together, these findings suggest that the aberrant function of *BSN* and *PCLO* are associated with neuropsychiatric disorders.

In our molecular genetic study series of psychiatric disorders, we conducted NGS analysis for families with singleton or multiplex affected members to search for their genetic underpinnings. We report the identification of three private rare pathogenic mutations of *BSN* and *PCLO* in three families with schizophrenia and bipolar disorder, respectively.

## 2. Materials and Methods

### 2.1. Subjects

All of the subjects were residents of Taiwan. We recruited families diagnosed with schizophrenia or bipolar disorder from the hospital. The Review Board of the Chang Gung Memorial Hospital-Linkou approved this study with the approval number 201801385A3. Each subject signed an informed consent form after a full explanation of this study. They received interviews and reviews of medical records to collect clinical information. Their psychiatric diagnoses were according to the criteria of the DSM-5 (Diagnostic and Statistical Manual of Mental Disorder—5th edition). They also donated blood for the preparation of genomic DNA to conduct NGS and Sanger sequencing.

### 2.2. Next Generation Sequencing Analysis

We conducted whole-exome sequencing (WES) using the Illumina HighSeq2000 platform and whole-genome sequencing (WGS) using the Illumina NovaSeq6000 platform (Illumina, San Diego, CA, USA) following the standard protocols provided by the manufacturer. After a quality check, the raw sequencing data were aligned to the human reference genome build hg19/GRch37. We then used SAMtools and a Genome Analysis Tool Kit to refine the local alignment and generate a variant calling file (VCF) for each subject. Variants were further annotated, filtered, and analyzed under different inheritance models, including autosomal-dominant, autosomal-recessive, X-linked, and de novo mutations. We performed bioinformatics and family analyses using SeqLab software (ATgenomics, Taipei, Taiwan). The NGS data are available upon request of the corresponding author.

### 2.3. Sanger Sequencing

We designed primer pairs to obtain amplicons covering the mutations by polymerase chain reaction (PCR) to verify the authenticity of mutations identified from NGS analysis. The primer sequences, optimal annealing temperature, and amplicon size are listed in Table 1. We used the forward primer for sequencing. In brief, we performed 30 cycles of PCR in a 20 μL mixture containing 100 ng of DNA, 1 μM of each primer, 1X buffer, 0.25 mM of dNTP, and 0.5 U of Power Taq polymerase (Genomics, New Taipei City, Taiwan). An aliquot of the amplicon was purified and subjected to Sanger sequencing using a BigDye Terminator Kit v3.1 (Applied Biosystems, Foster, CA, USA).

### 2.4. Bioinformatics Analysis

We checked the frequency of the mutation in the dbSNP (https://www.ncbi.nlm.nih.gov/snp/) (accessed on 9 October 2021) and the Taiwan Biobank (https://taiwanview.twbiobank.org.tw/index) (accessed on 9 October 2021). We assessed each mutation’s functional impact using Polyphen-2 (http://genetics.bwh.harvard.edu/pph2/index.shtml) (accessed on 9 October 2021), SIFT (https://sift.bii.a-star.edu.sg/) (accessed on 9 October 2021), and PROVEAN (http://provean.jcvi.org/index.php) (accessed on 9 October 2021).

## 3. Results

### 3.1. Identification of the R1087Q Mutation of BSN

**Figure 1:**
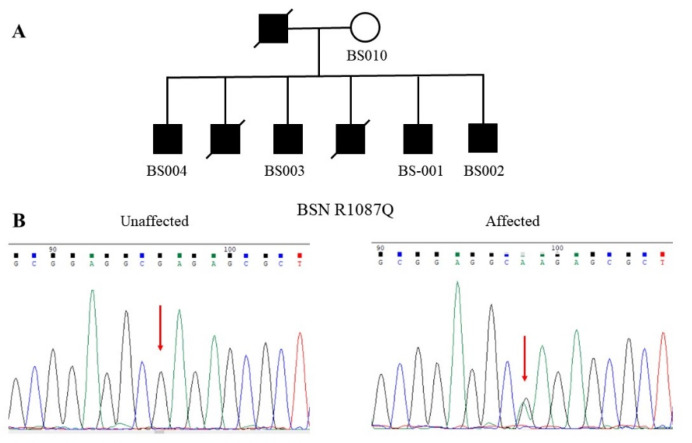
A multiplex family of schizophrenia with the R1087Q mutation of the *BSN* gene. (**A**) The pedigree shows the dominant inheritance of schizophrenia in this family. Only five members of this family were available for study. (**B**) Chromatograms show the wild-type G homozygote at the nucleotide position 49690249 of the *BSN* gene in the unaffected mother. At the same time, the other four patients carried the G-to-A heterozygous mutation of the *BSN* gene.

Figure 1A shows the pedigree of the families with multiple affected members diagnosed with schizophrenia. Schizophrenia was transmitted in this family in an apparent dominant inheritance. Three affected patients, including the father and his two children, had passed away due to medical illnesses. All of the living patients had an unremarkable birth history and normal psychosocial development before their psychotic symptoms, and they did not have an intellectual disability or neurological disease. The onset of schizophrenia in this family began from 17 to 21 years of age.

The WES analysis identified a G-to-A substitution at the nucleotide position 49690249 of BSN at chromosome 3 in all four affected patients (BS001–BS004). This mutation was not detected in the unaffected mother (BS010). The authenticity of this mutation was verified by Sanger sequencing (Figure 1B). The G-to-A substitution led to an amino acid change from arginine to glutamine at codon 1087, designated R1087Q. The mutation was assigned rs200987266 in the dbSNP. We did not find this mutation in the Taiwan Biobank. Moreover, it is very rare in several public genome databases, including Allele Frequency Aggregator Project (ALFA), Trans-Omics for Precision Medicine Whole Genome Sequencing Project (TOPMED), and 1000 Genomes project (1000 Genomes) (Table 1). Bioinformatics analysis predicted that this mutation has a damaging effect on the function of BSN (Table 1).

### 3.2. Identification of the S1535L Mutation of PCLO

**Figure 2:**
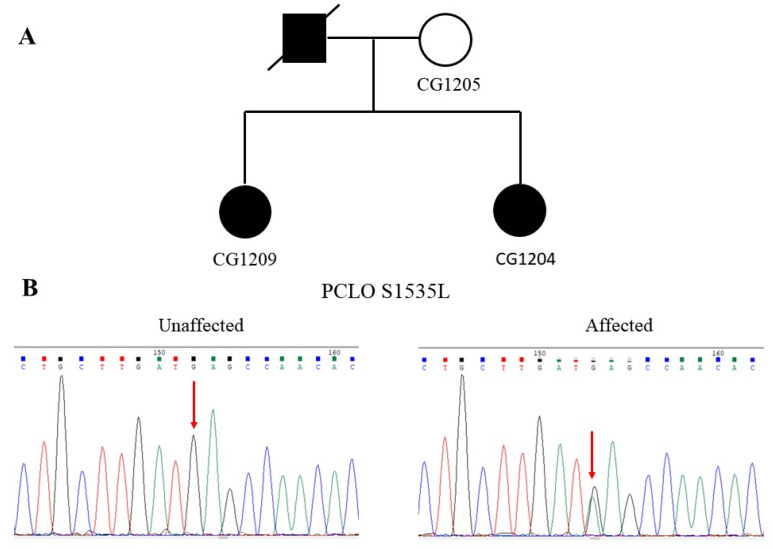
A family of bipolar disorder with the S1535L mutation of the *PCLO* gene. (**A**) The pedigree shows the dominant inheritance of bipolar disorder in this family. (**B**) Chromatograms show the wild-type G homozygote at the nucleotide position 82585665 of the unaffected mother’s PCLO gene. Simultaneously, both of the affected sisters carried the same G-to-A heterozygous mutation of the PCLO gene.

Figure 2A shows the pedigree of a family with two affected sisters diagnosed with bipolar disorder. Both sisters had an unremarkable birth history and normal psychosocial development before the onset of their mental illness. The elder sister began her mental illness at 28 years old, while the younger sister had her mental symptoms from 38 years old. They did not have an intellectual disability or neurological symptoms. Their father was diagnosed with bipolar disorder before and committed suicide when he was 58 years old. The mother did not have a history or symptoms of mental illness.

Under the autosomal-dominant inheritance model, we identified a G-to-A substitution at the nucleotide position 82585665 of *PCLO* at chromosome 7 in both sisters (CG1204 and CG1209) in the WGS analysis. This mutation was not present in their unaffected mother (CG1205). The authenticity of this mutation was verified by Sanger sequencing, as shown in Figure 2B. The G-to-A substitution resulted in an amino acid change from serine to leucine at the codon 1535, designated S1535L. The mutation was assigned rs757482765 in the dbSNP. This mutation was not found in the Taiwan Biobank, either. Moreover, the allele frequency of this mutation was very rare in several public genome databases, including Allele Frequency Aggregator Project (ALFA), Trans-Omics for Precision Medicine Whole Genome Sequencing Project (TOPMED), and 1000 Genomes project (1000 Genomes) (Table 1). Bioinformatics analysis predicted that this mutation is detrimental to the function of *PCLO* (Table 1).

### 3.3. Identification of the H5142R Mutation of PCLO

**Figure 3:**
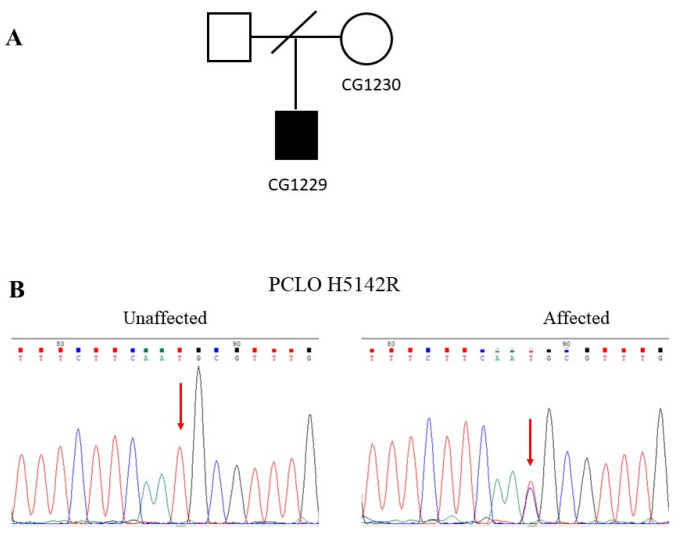
Family with the H5142R mutation of the PCLO gene. (**A**) A pedigree of family 3 with a patient diagnosed with paranoid schizophrenia. (**B**) Chromatograms show the wild-type T homozygote at the nucleotide position 82387895 of the unaffected mother’s PCLO gene. At the same time, the patient was a heterozygote of the T-to-C mutation of the PCLO gene.

Figure 3A shows the pedigree of a simplex family with a patient diagnosed with schizophrenia. The patient had an unremarkable birth history and normal psychosocial development before. He manifested psychotic symptoms such as auditory hallucinations, the idea of reference, and delusions of persecution when he was 27 years old. He was diagnosed with paranoid schizophrenia. He did not have an intellectual disability or neurological deficits. His parents had divorced, and we could not obtain clinical information or a DNA sample from his father for study.

In the WGS analysis, we identified a T-to-C substitution at the patient’s nucleotide position 82387895 of PCLO at chromosome 7 (CG1229). We did not detect this mutation in his mother (CG1230). The authenticity of this mutation was verified by Sanger sequencing (Figure 3B). The T-to-C substitution changed the amino acid histidine to arginine at the codon 5142, designated H5142R. Moreover, the mutation was assigned rs141283244 in the dbSNP. The allele frequency of this mutation was 0.008 in the Taiwan Biobank. In contrast, the allele frequency of this mutation was very rare in several public genome databases, including Allele Frequency Aggregator Project (ALFA), Trans-Omics for Precision Medicine Whole Genome Sequencing Project (TOPMED), and 1000 Genomes project (1000Genomes) (Table 2). Bioinformatics analysis predicted that this mutation is deleterious to the function of *PCLO* (Table 2).

## 4. Discussion

In this study, we identified a rare missense mutation (R1087Q) of *BSN* in a multiplex family of schizophrenia that consisted of seven patients, including the affected father and his six affected children. This mutation was found in four living brothers, presumably transmitted from their deceased father. Moreover, the mutation was likely present in the other two deceased brothers who were also diagnosed with schizophrenia before. In the second family, we identified a rare missense mutation (S1535L) of *PCLO* in two sisters diagnosed with bipolar disorder. This mutation was presumably transmitted from their father, who also suffered from bipolar disorder and suicide. In the third family, we identified another missense mutation (H5142R) of *PCLO* in a patient diagnosed with schizophrenia. We cannot determine whether this mutation was a de novo mutation or transmitted from his father because we could not obtain his DNA for study.

Rare missense mutations of *BSN* have been reported to cause progressive supranuclear palsy-like syndrome, a rare neurodegenerative disease [21]. Notably, our patients in this family presented with schizophrenia symptoms only; they did not have neurological symptoms such as seizures, dementia, or parkinsonism symptoms [21]. To the best of our knowledge, we are the first to report the *BSN* missense mutation in patients with schizophrenia. We suggest that mutations of *BSN* may have pleiotropic clinical effects, and schizophrenia may be one of the clinical manifestations of *BSN* mutations. The detection of R1087Q of *BSN* in the schizophrenia family suggests that *BSN* might be a risk gene for schizophrenia.

Several genetic studies have shown that SNPs of *PCLO* are associated with major depression, bipolar disorder, and drug abuse [23,24,25,26,27,28], while rare mutations of *PCLO* are associated with intellectual disability and pontocerebellar hypoplasia type III [29,30]. Furthermore, differential expression of the *PCLO* gene transcript has been reported in the postmortem brains of patients with bipolar disorder [27] and patients with schizophrenia [31]. Together, these studies imply the involvement of *PCLO* in the pathogenesis of neurodevelopmental disorders, affective disorders, and schizophrenia. Further functional studies are needed to address this association. The present study identified two missense mutations of *PCLO* in two families affected by bipolar disorder and schizophrenia, respectively. Notably, our patients did not have neurological symptoms or intellectual disability as described in other patients with rare *PCLO* mutations [29,30]. To the best of our knowledge, we are also the first to report the rare missense mutations of *PCLO* in patients with bipolar disorder and schizophrenia. Our findings provide further genetic evidence to support the involvement of *PCLO* in the pathogenesis of schizophrenia and bipolar disorder.

The molecular mechanisms underlying schizophrenia and bipolar disorder of these three rare mutations of *BSN* and *PCLO* identified in this study remain to be elucidated. Nevertheless, previous in vitro and animal studies may provide some insights. D-amino acid oxidase (DAAO) is an enzyme that catalyzes the oxidation of D-serine, a co-agonist of the N-methyl-D-aspartate receptor (NMDAR). As hypofunction of the NMDAR-mediated signaling pathway has been considered a molecular mechanism underlying schizophrenia [32], studies have shown reduced levels of D-serine in the cerebrospinal fluid and serum [33,34,35,36] and increased protein levels and activities of DAAO in patients with schizophrenia [35,37,38]. These findings suggest that increased DAAO activity may lead to reduced levels of D-serine that consequently result in hypofunction of NMDAR. Popiolek and colleagues conducted an immunoprecipitation of DAAO using rat brains. They found that both the BSN and PCLO proteins robustly interact with DAAO [39]. Additionally, they found that the BSN protein colocalizes with DAAO in the synaptic junction. Meanwhile, enzyme assays showed that the BSN protein significantly inhibits the DAAO activities. Based on these findings, we speculate that the *BSN* mutation found in this study may lead to disinhibition of DAAO, and the increased activity of DAAO may reduce the levels of D-serine in the brain, resulting in hypofunction of NMDAR-signaling. To test this speculation, further studies of protein–protein interactions between the BSN mutation and the PCLO and the DAAO can help address this issue. Moreover, measurement of the activity of DAAO in the blood of patients would help prove this speculation.

Furthermore, in a recent study, Nitta and colleagues reported that mice with suppression of *PCLO* expression in the medial frontal cortex showed schizophrenia-like behavioral impairments, including enhanced locomotor activity, impaired auditory prepulse inhibition, and cognitive dysfunction. Antipsychotic risperidone can partially improve these impairments in their study. [40]. Furthermore, the *PCLO*-suppressed mice showed increasing stress vulnerability when under mild social defeat stress. Their neurophysiological experiment in this animal model suggests that *PCLO* suppression in the medial frontal cortex may reduce neuronal connectivity between the medial frontal cortex and dorsal lateral striatum and cause schizophrenia-like behavioral impairments [40].

In conclusion, we identified three private rare pathogenic mutations in two PCM genes, *BSN* and *PCLO*, in three families with schizophrenia and bipolar disorder. Our results increase the genetic and allelic heterogeneity of schizophrenia and bipolar disorder and suggest that *BSN* and *PCLO* may be considered risk genes for schizophrenia and bipolar disorder. Our study also suggests that the aberrant function of PCM may contribute to the pathogenesis of schizophrenia and bipolar disorder.

## Figures and Tables

**Table 1:** Primer sequences, optimal annealing temperature (Ta), and amplicon size of Sanger sequencing for verification of the mutations identified in this study.

Mutation	Forward Primer (5′-3′)	Reverse Primer (5′-3′)	Ta (°C)	Size (bp)
BSN R1087Q	TGCGGGAGGAAGAGGAGCTGCTT	GGAGCGGTGTAGCTCCTCCATCT	60	227
PCLO S1535L	CTTCTGGCTCTCAGTACTGC	CCTTCCAGCAAGGACCATAA	60	318
PCLO H5142R	CCCACTCTTATGTTTGCCTCTC	GACCTTGATTGGTGAAGCCTG	60	214

**Table 2:** Location, frequency, and in-silico functional prediction of the three mutations identified in this study.

Gene Name and dbSNP Number	Location	Allele Frequency				Functional Prediction		
		Taiwan Biobank	ALFA	TOPMED	1000 Genomes	PROVEAN (Score)	PolyPhen-2 (Score)	SIFT (Score)
BSN rs200987266	NC_000003.11:g.49690249G > A NM_003458.3:c.3260G > A NM_003458.3:p.Arg1087Gln	0	0	0.000008	0.0002	Deleterious (−3.16)	Probably damaging (1.000)	Damaging (0.000)
PCLO rs757482765	NC_000007.13:g.82585665G > A NM_033026.5:c.4604C > T NM_033026.5:p.Ser1535Leu	0	0	0.000032	0.000056	Deleterious (−2.67)	Probably damaging (0.999)	Damaging (0.002)
PCLO rs141283244	NC_000007.13:g.82387895T > C NM_033026.5:c.15425A > G NM_033026.5:p.His5142Arg	0.00824	0	0.000191	0.000229	Deleterious (−3.27)	Probably damaging (0.996)	Damaging (0.002)

ALFA: Allele Frequency Aggregator Project; TOPMED: Trans-Omics for Precision Medicine Whole Genome Sequencing Project; 1000Genomes: 1000 Genomes project; PROVEAN: Protein Variation Effect Analyzer; PolyPhen-2: Polymorphism Phenotyping v2; SIFT: Sorting intolerant from tolerant.

## Data Availability

The data presented in this study are available on request from the corresponding author. The data are not publicly available due to privacy.
